# Rethinking domain adaptation for machine learning over clinical language

**DOI:** 10.1093/jamiaopen/ooaa010

**Published:** 2020-04-13

**Authors:** Egoitz Laparra, Steven Bethard, Timothy A Miller

**Affiliations:** o1 School of Information, University of Arizona, Tucson, Arizona, USA; o2 Computational Health Informatics Program, Boston Children’s Hospital, Boston, Massachusetts, USA; o3 Department of Pediatrics, Harvard Medical School, Boston, Massachusetts, USA

**Keywords:** machine learning, natural language processing, domain adaptation, shared resources

## Abstract

Building clinical natural language processing (NLP) systems that work on widely varying data is an absolute necessity because of the expense of obtaining new training data. While domain adaptation research can have a positive impact on this problem, the most widely studied paradigms do not take into account the realities of clinical data sharing. To address this issue, we lay out a taxonomy of domain adaptation, parameterizing by what data is shareable. We show that the most realistic settings for clinical use cases are seriously under-studied. To support research in these important directions, we make a series of recommendations, not just for domain adaptation but for clinical NLP in general, that ensure that data, shared tasks, and released models are broadly useful, and that initiate research directions where the clinical NLP community can lead the broader NLP and machine learning fields.

## INTRODUCTION

As developers and maintainers of the open-source Apache cTAKES clinical natural language processing (NLP) software, one of the most common questions we get from new users is “Why didn’t cTAKES correctly find phenomenon X in my data?” The problem is almost always that cTAKES’s statistical model for phenomenon X is trained on data that does not have examples like those in the user’s data. Inevitably, the next question is, “How can I add this example?” to which the answer is a politer version of, “Machine learning doesn’t work that way.” But maybe it should.

The standard machine learning answer to getting a model that was trained on data from one domain to perform well on data from another domain is *domain adaptation*. These algorithms are designed to work regardless of the definition of *domain*, whether it be adapting from one medical specialty to another, adapting from one institution’s formatting standards to another, etc. In the clinical domain, it has been widely documented that without domain adaptation, performance of Clinical NLP systems degrades seriously in the face of new domains (see Supplementary Appendix A). The vision of applying domain adaptation is therefore attractive, but the data sharing restrictions in the clinical domain present significant obstacles to this vision. Even datasets created for the express purpose of sharing can be difficult to work with, requiring IRB approvals, data use agreements (potentially requiring legal review by the receiving site), and human-subjects training for all users. There are several instances where datasets created for shared tasks had to be withdrawn due to institutional cold feet. In other cases, when funding dries up, since it is not possible to simply dump the data into the public domain, the data essentially disappears. The difficulties presented by clinical text have not received proper attention in the NLP literature. For example, we found more than 60 publications on domain adaptation in the most relevant NLP venues of the last 3 years, of which just 15 cover clinical domain and only one^1^ mentions the data sharing restrictions that are fundamental to this domain.

In the remainder of this perspective, we first present a taxonomy of domain adaptation methods which carefully considers data sharing constraints and demonstrates that, while a wide variety of domain adaptation algorithms have been proposed, the vast majority do not apply in realistic clinical settings. We therefore present a series of recommendations designed to guide machine learning research in directions that satisfy the fundamental data privacy needs of clinical records.

## A taxonomy of domain adaptation methods

Domain adaptation techniques can be conceptually divided into *supervised domain adaptation*, where some of the target data is labeled, and *unsupervised domain adaptation*, where none of the target data is labeled. The supervised version is uncommon in the clinical domain since creating new labels usually requires a rare combination of linguistic and medical knowledge. But more critically, this classical division says nothing about data sharing, and many supervised and unsupervised domain adaptation techniques assume that they have simultaneous access to data from both the source and target sites. This assumption is unrealistic in the clinical domain, where most datasets cannot be shared across institutions, and even datasets created with the intention of sharing can carry onerous restrictions. Some techniques exist for training supervised models on data from multiple non-sharable sources (eg, *federated learning*^2^ or *split learning*^3^ where a single model is trained collectively by multiple devices), but they assume that annotation expertise is easily available for each new domain, which is not true for clinical NLP problems.

We therefore propose a conception of the space of possible domain adaptation methods that takes into consideration the above factors. We consider three possibilities for what the source site shares:

1. *Labeled text*: the target site can see everything at the source site.
2. *Labeled feature vectors*: the raw text is not shared but features extracted from the raw text and the labels for those feature vectors are. (This typically precludes neural network models which learn features from raw text.)
3. *Trained models*: only a final model is shared.

We consider two possibilities for what type of data is available at the target site:

1. *Raw text*: a large amount of unlabeled target site data.
2. *Labeled text*: a small amount of target site data, labeled in the same way as the source site, along with a larger amount of raw text as above.

We consider two possibilities for what the target site might share back with the source site:

1. *Nothing*: no data are shared.
2. *Models*: statistical models of the target data are shared with the source. This is relevant only when the source shares no labeled data, since if the source shares labeled data, all models can be constructed at the target site.

We multiply out the space of these possible adaptation methods, as shown in Table 1.

**Table 1.** A proposed categorization of the space of domain adaptation algorithms

Source shares	Target has	Target shares	Best methods
Labeled text	Labeled text	–	Neural feature augmentation^5^ Parameter transfer^7–9^ Prior knowledge^10^ Instance weighting and selection^11^^,^^12^
	Raw text	–	Neural feature correspondence learning^14^ Re-training embeddings^19^ Bootstrapping^20^^,^^21^ Adversarial training^22^ Auto-encoders^16–18^
Labeled features	Labeled text	–	Feature augmentation^6^
	Raw text	–	Feature correspondence learning^13–15^
Trained Models	Labeled text	–	Fine-tuning^23^^,^^24^ Adaptive off-the-shelf^25^
		Models	–
	Raw text	–	Online self-training^21^
		Models	Pseudo in-domain data selection^26^

*Notes:* It is assumed that there is always labeled data available in the source domain. “Source shares” describes what the source site is able to share with the target site. “Target has” describes what data are available at the target site. “Target shares” describes what the target site is able to share with the source site. “Methods” gives names for the types of methods in each configuration, and citations to examples of such work

The first four rows represent the vast majority of domain adaptation research. We cite some of the most popular algorithms and describe them briefly in this paragraph, but there are hundreds more publications in these areas (see the survey in ref.^4^) When the source can share data and the target has labeled data, domain adaptation is at its most effective; some approaches are *feature augmentation*,^5^^,^^6^ where the feature space is multiplied out to contain versions of each feature for the source, target, and shared domains; *parameter transfer*,^7–9^ where some parameters of the source and target models are shared and trained jointly; and *prior knowledge based*^10^ and *instance weighting and selection*,^11^^,^^12^ where distributions learned from the labeled target data form a prior either to train the model or to weight or select the proper examples in the training set. However, that setting is the least realistic for the clinical setting. A somewhat more realistic setting for clinical data is where the source can share data but the target has no labeled data, encompassing, for example, the i2b2 and n2c2 shared tasks. Methods for this setting are not as effective but there is substantial research in this direction: *feature correspondence learning*^13–15^ and *auto-encoders*,^16–18^ where a shared feature space between source and target domains is learned; *re-training embeddings*,^19^ where the first layers of a neural network model are pre-trained on unlabeled data from both the source and target domains; *bootstrapping*,^20^^,^^21^ where a source-domain-trained model is re-trained on its own predictions in the target domain combined with the source domain data, and *adversarial learning*^22^; where a model is trained to be unable to distinguish the source and target domains while still performing well on the source domain training data.

The last two rows of the table encompass the part of the space that is critical for clinical NLP research, where the source cannot share labeled data or features. Unlike the first four rows, which list just the most representative methods from the literature, these rows are an exhaustive list of all research we could find in these areas. As the table illustrates, there is little research to date on such techniques. Examples include *fine-tuning* (common in single-domain settings, but rarely studied as a domain adaptation technique), where a model is pre-trained on the source data, then transferred to the target domain for continued training; *adaptive off-the-shelf* framework, where the model is treated as a black-box and the adaptation is performed at the output level; *online self-training*, where the model is re-trained on only its own predictions in the target domain; and *pseudo in-domain data selection*, *where* instances in the source data are selected according to the perplexity of a language model pre-trained in the target domain. Each of these approaches have significant drawbacks, and many have not been evaluated on any clinical data. We thus see the urgent need for further work in these areas of domain adaptation research if we want our machine learning models to be usable in the clinical setting.

## Prescriptions

To address the urgent need for machine learning methods that can be applied under the data sharing constraints of the clinical domain, we assert that generalizable methods should be at the forefront, not just a consideration of those focusing on domain adaptation research. In that spirit, we make the following recommendations that we believe should apply to *all* clinical NLP research:

1. *Datasets* of annotated clinical language should always be constructed from at least two different data distributions—if not different institutions then at least different parts of the same institution (intensive care unit, oncology, cardiology, etc.). This ensures that models trained using the annotations can be evaluated for their robustness across the different data partitions. This change would have a major positive impact on research in generalizable methods of all sorts but is of course a necessary prerequisite to the constrained form of domain adaptation we emphasize here.
2. *Shared tasks*, where participants develop research systems for a task and compare them on a shared dataset, should include scenarios where the full source data is not available. For example, a shared task could have two tracks—one traditional generalizability track with labeled source data and unlabeled target data, and another where the only available information from the source is a trained model from a standard toolkit (eg, BERT). This ensures that the performance reported by such shared tasks is a meaningful estimate of future performance on new clinical data, under different possible data sharing constraints.
3. *Software* containing machine learning models should explicitly describe the datasets used to train it, especially if the data is not part of a shared task or publicly available. We also encourage software design that provides explicit application programming interfaces (APIs) to domain adaptation algorithms that articulate the data sharing assumptions and simplify the process of adapting the distributed models to new domains.
4. *Users* of clinical NLP software should make sure they know what data a system has been trained on. Even when the original data seems compatible with the user’s own data, users should carefully inspect the system’s output. If the model performs poorly, in addition to reporting the problems to the developers, users should try whenever possible to find a source of shareable data that also demonstrates the problem.
5. *Researchers* in clinical NLP should treat domain adaptation, transfer learning, etc. as a first-class problem rather than a niche area. Research efforts should shift towards methods in the bottom quarter of Table 1. This offers the opportunity for clinical NLP researchers to take the lead in an area which is underserved by methods in the general domain, and solve problems in the most realistic setting. The research community should create centralized repositories for sharing trained models, so that even internally created, non-sharable datasets can provide community benefit.
6. *Funders* who want clinical NLP research they fund to have maximum impact should consider novel mechanisms that would allow for the software development recommendations described above, especially the implementation of APIs for adapting models in the face of data sharing constraints. There is an incentive misalignment, where individual researchers are reluctant to spend grant money on activities that do not advance their personal scientific aims, but agencies would like the tools developed with their funding to be as robust as possible. Relatively small amounts of funding for these activities could contribute greatly to the missions of the agencies that typically fund clinical NLP research. Data sharing policies should take into account the difficulty of sharing text data and promote and reward the sharing of statistical models trained on such data.

The field of clinical NLP should treat this as an opportunity to take the lead on an important problem that is not well-studied in general domain machine learning. The unique data sharing challenges of the clinical domain make a perfect testbed for this research, and the clinical NLP community has a strong motivation to address these challenges. This is an exciting opportunity for our research community to develop innovative new machine learning methods that potentially extend even beyond the clinical domain.

## FUNDING

Research reported in this publication was supported by the National Library of Medicine of the National Institutes of Health under Award Number R01LM012918. The content is solely the responsibility of the authors and does not necessarily represent the official views of the National Institutes of Health.

## AUTHOR CONTRIBUTIONS

All authors contributed to the conception, writing, and editing of the manuscript. EL performed the literature review.

## SUPPLEMENTARY MATERIAL

Supplementary material is available at *Journal of the American Medical Informatics Association* online.

## CONFLICT OF INTEREST STATEMENT

None declared.

## Supplementary Material

ooaa010_Supplementary_Data
