# Supplementary material for: Rethinking domain adaptation for machine learning over clinical language
Source: JAMIA Open. 2020 Apr 13;3(2):146–50. doi: 10.1093/jamiaopen/ooaa010 (PMC7382626; doi:10.1093/jamiaopen/ooaa010)
Supplement: ooaa010_Supplementary_Data [file ooaa010_supplementary_data.docx]

**Appendix A**

Table 2: A sample of published work that demonstrated performance degradation when NLP systems were applied to new target domains. MIMIC=Medical Information Mart for Intensive Care, BIDMC=Beth Israel Deaconess Medical Center, ICU=Intensive care unit, PAD=Peripheral arterial disease, SGH=Seattle Group Health, UPMC=University of Pittsburgh Medical Center

|  | Source | | Target | |
| --- | --- | --- | --- | --- |
| Task | Domain | Performance | Domain | Performance |
| Sentence segmentation [27] | Mayo Clinic (Diverse note types) | 94.9 Acc [28] | MIMIC II [29] (BIDMC ICU) | 31.8 Acc |
| Constituency parsing [30] | Wall Street Journal | 89 F1 | GENIA (Medline abstracts) | 75 F1 |
| Negation detection [31] | Partners Healthcare, BIDMC (Discharge summaries) | 93.6 F1 | Mayo Clinic (PAD), SGH (Breast oncology) | 74.7 F1 |
| Temporal information extraction [32] | Mayo Clinic (colon cancer) | 80 F1 (times)  92 F1 (events)  48 F1 (relations) | Mayo Clinic (brain cancer) | 59 F1 (times)  76 F1 (events)  34 F1 (relations) |
| Dependency parsing[33] | Wall Street Journal | 90 F1 | Medline abstracts (Cancer oncology) | 84 F1 |
| Coreference resolution [34] | Mayo Clinic (Colon cancer) | 55.3 CoNLL | UPMC (Breast cancer) | 52.5 CoNLL |
